# Acceptability and Usefulness of a Dyadic, Tailored, Web-Based, Psychosocial and Physical Activity Self-Management Program (TEMPO): A Qualitative Study

**DOI:** 10.3390/jcm9103284

**Published:** 2020-10-13

**Authors:** Sylvie D. Lambert, Lindsay Rosamond Duncan, Janet Ellis, Jamie Lynn Schaffler, Ekaterina Loban, John Wellesley Robinson, Nicole Culos-Reed, Andrew Matthew, Karissa Clayberg, Daniel Santa Mina, Lauren Goldberg, Phil Pollock, Simon Tanguay, Wassim Kassouf, Paramita Saha-Chaudhuri, Stuart Peacock, Anne Katz

**Affiliations:** 1 Ingram School of Nursing, McGill University, Montreal, QC H3A 2M7, Canada; 2 St. Mary’s Research Centre, Montreal, QC H3T 1M5, Canada; jamie.schaffler@mail.mcgill.ca (J.L.S.); ekaterina.loban@mail.mcgill.ca (E.L.); karissaclayberg@gmail.com (K.C.); 3 Department of Kinesiology and Physical Education, McGill University, Montreal, QC H2W 1S4, Canada; lindsay.duncan@mcgill.ca; 4 Department of Psychiatry, University of Toronto, Toronto, ON M5T 1R8, Canada; janet.ellis@sunnybrook.ca; 5 Sunnybrook Health Sciences Centre, Toronto, ON M4N 3M5, Canada; lauren.goldberg@sunnybrook.ca; 6 Department of Family Medicine, McGill University, Montreal, QC H3S 1Z1, Canada; 7 Department of Psychology, University of Calgary, Calgary, AB T2N 1N4, Canada; john.robinson@albertahealthservices.ca; 8 Department of Oncology, Cumming School of Medicine, University of Calgary, Calgary, AB T2N 4N1, Canada; nculosre@ucalgary.ca; 9 Faculty of Kinesiology, University of Calgary, Calgary, AB T2N 1N4, Canada; 10 Tom Baker Cancer Centre, Calgary, AB T2N 4N2, Canada; 11 Departments of Surgery and Psychiatry, Faculty of Medicine, University of Toronto, Toronto, ON M5S 1A8, Canada; andrew.matthew@uhn.ca; 12 Princess Margaret Cancer Centre, Toronto, ON M5G 2C1, Canada; daniel.santamina@uhn.ca; 13 Faculty of Kinesiology and Physical Education, University of Toronto, Toronto, ON M5S 2W6, Canada; 14 Department of Clinical Trials & Clinical Research, BC Cancer—Victoria, Victoria, BC V8R 6V5, Canada; philip.pollock@bccancer.bc.ca (P.P.); speacock@bccrc.ca (S.P.); 15 Department of Surgery, Faculty of Medicine, McGill University, Montreal, QC H3G 2M1, Canada; simon.tanguay@mcgill.ca; 16 Division of Urology, McGill University Health Centre, Montreal, QC H4A 3J1, Canada; wassim.kassouf@muhc.mcgill.ca; 17 Department of Mathematics and Statistics, University of Vermont, Burlington, VT 05401, USA; paramita.chaudhuri@mcgill.ca; 18 Faculty of Health Sciences, Simon Fraser University, Burnaby, BC V5A 1S6, Canada; 19 College of Nursing, University of Manitoba; Winnipeg, MB R3T 2N2, Canada; anne_katz@umanitoba.ca; 20 The Dr. Ernest W. Ramsey Manitoba Prostate Centre, CancerCare Manitoba, Winnipeg, MB R3E 0V9, Canada

**Keywords:** prostate cancer, cancer survivorship, cancer rehabilitation, self-management, caregivers, dyadic intervention

## Abstract

Caregivers of men with prostate cancer report high burden, and there is a need to develop cost-effective programs to support them in their roles. This study reports on the acceptability of a dyadic, **T**ailored, w**E**b-based, psychosocial and physical activity (PA) self-**M**anagement **P**r**O**gram called TEMPO. TEMPO was accessed by a convenience sample of 19 men with prostate cancer and their caregivers (*n* = 18), as well as six health care professionals (HCPs). User feedback was gathered via semi-structured qualitative interviews. Data were analyzed using thematic analysis. Most dyads were satisfied with TEMPO, particularly with the dyadic feature of TEMPO, the focus on goal setting to integrate self-management, and the extensive health library. The patients and caregivers motivated each other as they worked through TEMPO. Most goals to achieve during TEMPO pertained to increasing PA, followed by learning physical symptom management. One unanticipated benefit of TEMPO for the dyads was improved communication. HCPs agreed that TEMPO was a novel approach to online cancer self-management and they echoed the benefits reported by dyads. Key suggestions for improving TEMPO were to reduce repetition, tailor content, add more exercise ideas, and have more printing options. This study provides a strong foundation on which to plan a larger trial.

## 1. Introduction

Despite the improvement in survival rates, a prostate cancer diagnosis still elicits negative reactions and confronts both patients and their caregivers with a wide range of physical and psychosocial challenges [1,2,3]. Though caregivers’ support reduces the demands on the health care system [4], and positively impacts their loved ones’ illness adjustment, this support comes at a particularly high cost to caregivers’ own health [1,2,5,6,7]. Cancer caregivers (especially females [4]) have been found to be at higher risk of physical and emotional burden than those who care for individuals with diabetes or frail elders [5]. Studies suggest that a third of cancer caregivers report clinically significant levels of anxiety 6 months post patient diagnosis, and continue to be anxious for up to 5 years [6,7,8]. This prevalence exceeds the anxiety rate reported by the patients themselves [9] and population norms [6]. In addition to the emotional burden, caregivers are exposed to social, financial, and physical burdens, which pose further risks to their health [1]. One study found that 43% of caregivers experienced a steady decline in (or chronically below the population norm) physical health in the first 5 years following the patients’ diagnosis [10].

In light of caregivers’ challenges and the importance of them being able to maintain their vital roles, a number of interventions (mostly based in the principles of psycho-education and self-management) have been developed to support caregivers and improve their quality of life (QOL) [11,12,13]. Trials have substantiated the efficacy of these interventions in reducing caregivers’ burden, and improving their ability to cope, relationship functioning, and aspects of QOL [12,13]. Nonetheless, these interventions often neglect the caregivers’ physical health needs, and thus have limited (or no) impact on their physical functioning.

With the recognition of the physical and psychological benefits of physical activity (PA) for the general population [14], regular PA now represents an important self-management strategy to help caregivers maintain their health at the level required to successfully perform their vital roles [15]. Swartz and Keir [16] found that cancer caregivers want to engage in PA programs to help them reduce their burden. However, more rigorous trials are needed to support this self-management approach among caregivers [15]. In addition, most studies on caregiver PA programs target the individual caregiver, missing an opportunity to involve the caregiver–patient dyad. It has been suggested that when interventions engage patients and caregivers (as a dyad), important synergies are achieved that can contribute significantly to each person’s well-being outcomes [17,18], and that have a positive impact on intervention adherence [19].

To date, the existing research [11] highlights gaps in the ability to effectively deliver sustainable caregiver interventions, emphasizing the urgency to find alternative modes of delivery, such as a self-directed (or home-based) format [20,21,22]. Two dyadic, self-directed interventions for caregivers have been published: Coping-Together [11,23,24] and FOCUS [25,26]. These interventions provided evidence of the feasibility and/or efficacy; however, they lacked the integration of health promotion strategies such as PA. Recently, Cuthbert et al. [27] addressed this gap with the development of the structured, center-based RECHARGE intervention, which has been found to be efficacious on a number of psychosocial outcomes [27]. Only FOCUS [25,26] is available in a web-based format (but does not address PA). Web-based interventions have been proposed as a convenient, effective, and economical approach for delivering support to large populations [28], and thus represent a particularly effective strategy for providing cancer support to dyads.

To address the gap in the current literature, we developed the first dyadic, **T**ailored, w**E**b-based, psychosocial and PA self-**M**anagement **P**r**O**gram (TEMPO) for men with prostate cancer and their caregivers. As an individual’s acceptance of the technology is a strong predictor of future adoption, [29] the aim of this paper is to report on the acceptability of TEMPO to patients, caregivers, and health care professionals (HCPs) as well as its usability.

## 2. Methods

### 2.1. Design

This was a longitudinal, multi-center, qualitative study involving semi-structured interviews with prostate cancer patients and their caregivers, as well as HCPs. Qualitative data were supplemented with user-tracking information. Ethics approval was obtained from all sites.

### 2.2. Sample

**Figure 1:**
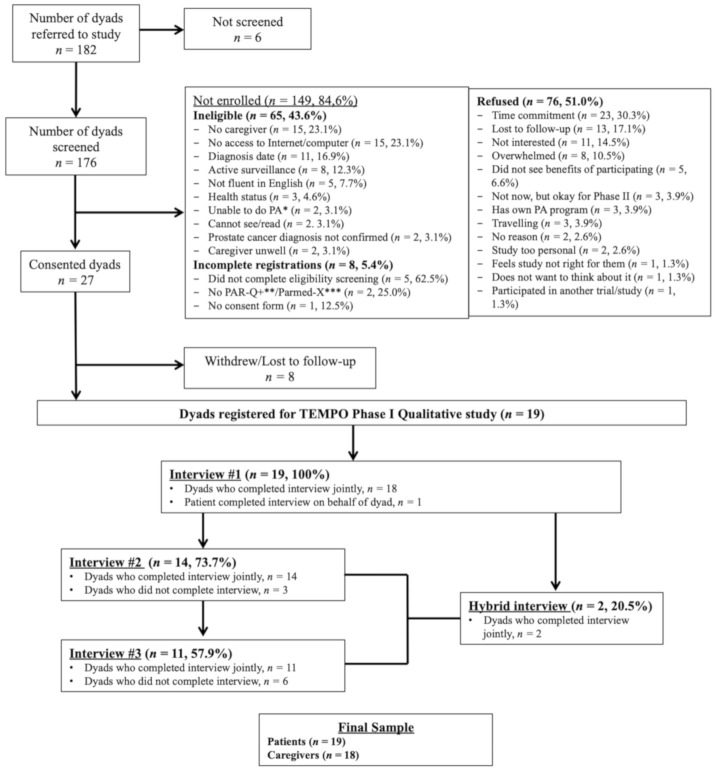
Flow diagram of a participant’s involvement in the qualitative assessment of TEMPO for dyads facing prostate cancer. Note: * Physical Activity (PA); ** Physical Activity Readiness Questionnaire for Everyone (PAR-Q+); *** Physical Activity Readiness Medical Examination (Parmed-X).

A convenience sample of 19 patients and their caregivers (*n* = 18) was recruited (see Figure 1). Patient inclusion criteria were: (a) confirmed prostate cancer diagnosis (localized or advanced) within the past two years, (b) a primary caregiver willing to participate, (c) undergoing or had undergone active treatment (excludes watchful waiting and active surveillance), and (d) having access to the Internet. Eligible caregivers were those identified by each patient as his primary source of support (regardless of relationship). Caregivers who were diagnosed with cancer in the previous year, receiving treatment for cancer, or had undergone any structured self-management program previously were excluded. Patients and caregivers also needed to be able to understand English (or have someone that could help them) and be free from medical contraindications to participate in moderate PA.

HCPs included the multidisciplinary oncology health provider team, managers, and representatives from community organizations. To participate, one of the following inclusion criteria had to be met: (a) providing frontline cancer care, (b) being in a leadership position at one of the participating centres, or (c) nominated by a community organization representing individuals with cancer and/or their caregivers.

When the interviews no longer provided new analytical information, it was determined that a reasonable exploration of participants’ opinions was achieved [30] and recruitment was stopped.

### 2.3. Recruitment Procedures

Participants were primarily recruited from four major cancer centres across Canada (June 2017–August 2018), whereby potential dyads were identified by clinicians, briefly introduced to the study, and then met with the on-site research assistant (RA) to receive further study information. If the caregiver was not present at the time of recruitment, the RA provided study information to the patient and obtained his verbal consent to follow up by phone to determine the caregiver’s interest. The link to the online consent form was given to interested dyads. Once consent was received, the participants were screened with the Physical Activity Readiness Questionnaire for Everyone (PAR-Q+) [31] to ensure that they were physically able to participate in the study. Patients or caregivers who answered ‘Yes’ to any of the PAR-Q+ questions were referred to a Certified Exercise Physiologist for further assessment. Community-based recruitment was also undertaken (e.g., advertisement in newspapers and recruitment through the TrueNTH lifestyle management website).

HCPs were identified through the team’s network and an online search of directories of health care centres. HCPs were initially approached by e-mail with a consent form. Interested HCPs were given the option of returning their consent form by e-mail or completing it online. Participating HCPs were asked to identify additional stakeholders (snowball sampling).

### 2.4. TEMPO

TEMPO (https://tempo.truenth.ca/) was designed to help dyads manage their psychosocial needs by learning new self-management strategies and increasing their PA level, mainly through walking and strength-based exercises, as a general health promotion strategy. Three theoretical frameworks guided the development of TEMPO:

- The Stress and Coping framework [32], whereby TEMPO aims to expand the dyads’ repertoire of active coping strategies.
- Bodenmann’s framework of dyadic coping [33], by focusing on enhancing positive dyadic coping as a means of increasing illness adjustment [34].
- Self-Efficacy Theory [35], through the integration of self-efficacy enhancing strategies of achievement of behavioural goals, behaviour modeling by similar others, and verbal persuasion.

**Figure 2:**
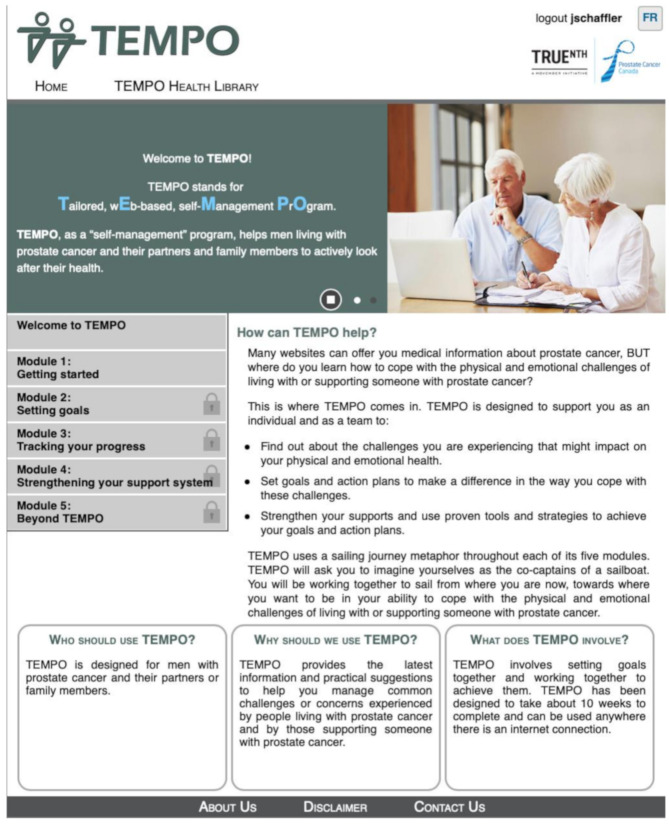
TEMPO landing page. This figure illustrates the navigational features of TEMPO including the introduction, the five modules dyads completed, and the health library [40].

TEMPO is a 10-week, interactive, web-based five module intervention, whereby a new module is released approximately every two weeks to pace dyads’ learning and avoid information overload [25]. A screenshot of the landing page is included in Figure 2. The modules include: (1) getting started (needs assessment), (2) setting goals and making action plans, (3) tracking progress, (4) identifying and strengthening support systems, and (5) self-managing after TEMPO. The modules were intentionally designed to focus on specific aspects of the behaviour change process with integrated persuasive technology techniques [36] such as primary task support, dialogue support, system credibility support, and/or leveraging social support. It was expected that patients and caregivers would initiate the modules together, but then progress at their own speed. Each dyad was encouraged to set two Specific, Measurable, Attainable, Relevant, Time-oriented and Together (SMARTT) goals, with at least one pertaining to PA. Each module specified online (e.g., interactive worksheets) and offline (e.g., engaging in PA) activities. By working through the modules, it was hoped that the dyads would feel more confident in using strategies demonstrated to be effective in addressing their psychosocial needs and develop the self-management skills necessary to meet the PA guidelines for aerobic (150 min of moderate-intensity exercise or 75 min of vigorous-intensity exercise or an equivalent combination) and strength (two to three weekly sessions that include exercises for major muscle groups) exercises [37,38,39]. Resistance bands and a pedometer were provided to each dyad to support their engagement in PA. Although exercise prescriptions were not issued, a factsheet in the TEMPO health library provided examples of light, moderate, and hard exercises that the dyads could do together (e.g., resistance band tricep kickbacks, alternating rows, etc.) and encouraged them to tailor their strength regimes to their fitness levels. Central to the design of TEMPO was the use of a sailing metaphor, to anchor the learning and change process in an easy to grasp analogy.

In addition, TEMPO included a health library incorporating 45 factsheets based on the most up-to-date evidence on self-management and PA (see Supplementary data S1 for both a figure of the TEMPO Health Library landing page, and a table detailing the topics of the 45 factsheets). The health library included seven sections: (a) communicating with your health care team, (b) treatment decision-making, (c) dealing with stress and worry, (d) supporting each other, (e) getting the support you need, (f) wanting to feel more fit and healthy, and (g) getting on top of symptoms. Once dyads identified their needs, they used the appropriate factsheets to get ideas for self-management strategies to address these and set their SMARTT goals accordingly. The health library content was mainly based on Coping-Together, previously developed by the team [11].

### 2.5. Data Collection

Once patients and caregivers submitted their consent forms, they were e-mailed the TEMPO log-in information, and asked to use it for 10 weeks. Throughout this period, the dyads were invited to participate in three semi-structured interviews (face-to-face or over the phone with an RA) to obtain their feedback as they worked through the modules. Interviews were conducted by RAs trained in using the interview guide and with experience in qualitative research. The interviews covered the perceived benefits of TEMPO, the acceptability of the dyadic features, accessibility, gaps, responsiveness, and appropriateness. Supplementary data S2 includes all three interview guides. A combined interview guide was developed for those dyads that could not participate in all three interviews. Of note, one couple felt more comfortable expressing themselves in their native tongue, and thus had an adult child translating all interviews. In addition to the interviews, user-tracking provided data on the number of logins and the length of sessions.

HCPs were provided with the log-in to TEMPO for three weeks and subsequently participated in one semi-structured interview conducted by an RA (face-to-face or over the phone) to discuss their perceptions of the program. All interviews were audio-recorded and transcribed verbatim. Field notes were not taken. Transcripts were not returned to the participants for comments or corrections.

### 2.6. Data Analysis

Thematic analysis was used to identify patterns within the data [41,42]. The coding outline was initially developed based on research aims and sections of the interview guide, including needs addressed by TEMPO, patterns of use, adherence, usefulness/benefits, and suggested improvements. Within this coding scheme, concepts from the aforementioned frameworks guiding the development of TEMPO were integrated. For example, within coding patterns of using TEMPO, attention was given to dyadic coping based on Bodenmann’s framework of dyadic coping [33]. Qualitative data analysis was carried out using NVivo (QSR International Pty Ltd, Version 12, Doncaster, Victoria, Australia). Transcripts were initially read and words, sentences, and/or sections related to the study aims were extracted either using pre-determined codes from the initial coding outline or new codes, if the coding outline did not reflect the data, with similar excerpts given the same code. All codes were then combined into categories and subcategories to reflect the main sections of the interview guide. The codes were then compared across interviews to identify similarities and differences, leading to the identification of themes. The first seven patient–caregiver transcripts were coded by two experienced qualitative RAs. The codes assigned by each RA were compared and discrepancies discussed. The coding structure was revised after each interview. The remaining 39 transcripts were coded independently by one of the RAs. In the results, “dyads” is used to describe themes expressed jointly by patients and caregivers, whereas “participants” is used if a single member of the dyad expressed the theme. All HCP transcripts were coded independently by two experienced qualitative RAs, and discrepancies in coding were discussed and resolved in team meetings.

### 2.7. Maintaining Research Quality

The consolidated criteria for reporting qualitative research framework were used to guide reporting [43]. The methods used to enhance rigor were: (a) transcribing interviews [44], (b) prolonged period of data collection [45], (c) letting participants guide the inquiry process [46], (d) clearly delineating the scope, (e) relating key findings to the literature [46], (f) using a clear coding procedure [47], (g) including quotes in the manuscript [48], and (h) keeping many drafts of the findings [45]. To further enhance rigor and maintain confidentiality, participant quotations included in the manuscript were given the following codes: participant group (PT= patient; CG = caregiver, HCP = healthcare provider), followed by the participant/dyad unique identifier(s), and the interview time point (1, 2, 3, or H = hybrid) from which the quote originated.

## 3. Results

### 3.1. Study Participants

The results are based on the analysis of 52 interviews over time, including 46 interviews across 19 dyads and six HCP interviews. Table 1 provides a detailed description of the socio-demographic characteristics of the 19 dyads. On average patients were in their early 60s, married, and living with their caregivers. Most patients had received their prostate cancer diagnosis in the early stages (*n* = 11) within the past 1–3 years (*n* = 10). The caregivers were exclusively female.

Six HCPs were recruited; of which most were female (*n* = 5), working in oncology either as managers (*n* = 2), or clinicians (*n* = 4). HCPs worked in the fields of nursing (*n* = 3), psychology/behavioral science (*n* = 1), social work (*n* = 1) and administration (*n* = 1), with two thirds having 9 or more years of experience (*n* = 4).

### 3.2. Relevance of TEMPO: Identification of Needs and Setting Goals

More than half of the dyads identified needing a lot of help with at least one need in Module 1, with most challenges identified pertaining to increasing PA levels and patient symptom management. In Module 2, the dyads then engaged in a process of narrowing down their needs to two SMARTT goals, with the majority setting dyadic goals pertaining to PA. These included exercising consistently, exercising safely, motivating each other, and finding a common exercise: “[T]he goals we’d set were to exercise and begin [...] being physically active, safely. And the second goal was really knowing how much physical activity we should do” (PT, 11023-12023-2). In addition to a dyadic goal, several patients and a few caregivers set an individual PA goal (see Table 2), including achieving a healthy weight, improving strength, and/or re-gaining energy and muscle mass. Although several participants reported high levels of fitness at the outset of TEMPO, their goal was to maintain their fitness levels. The few dyads reporting differences between the patient’s and the caregiver’s fitness levels set PA goals to balance their differences. Interestingly, exercise was not only viewed from the perspective of concrete physical benefits, but dyads chose PA as a self-management strategy (over other potential strategies) for physical symptoms and/or emotional concerns (e.g., mood changes, anxiety, stress). This explains why for many dyads both SMARTT goals pertained to PA.

When they got into the part talking about how exercise can […] help you with all of these things, then we thought, well let’s just […] put our-our goals as the exercise since you’re sending us this exercise stuff anyway. And-and let’s just focus on it and hopefully it-it’ll help us work through some of the other things (CG, 81018-82018-1).

A few patients set their SMARTT goals to learn self-management strategies to cope with symptoms, including anxiety, fatigue, or incontinence, dealing with erectile dysfunction, and controlling hypertension and/or diabetes. A couple of dyads set their SMARTT goals pertaining to improving communication with HCPs, including “obtaining more information to our answers from the doctor” (PT and CG, 11019-12019-1).

Half of the dyads acknowledged that they viewed their goals as dynamic and revised them as time progressed:

Caregiver: […] after we did module three, we went back and refined some of the things that we felt we’d already accomplished […].

Patient: […] we have to review our goals, but we may need to set new goals, and also revise the action plan. So, it’s not a static model (11019-12019-2).

The reasons for changing goals included: accomplishing initial goals sooner than anticipated, changing the type of PA due to challenges experienced, and making goals more realistic. Although most dyads set two SMARTT goals, several mentioned that there should be more explicit guidance on setting goals that addressed caregivers’ concerns or permit more than two goals, as goals tended to remain focused on patients.

### 3.3. Perceived Ease of Use of TEMPO

Most dyads were satisfied with TEMPO. The participants described their overall experience as “smooth” (31054-32054-H), “easy” (PT, 21023-22023-1), and were “glad we’re doing it” (PT, 11019-12019-2). They felt TEMPO was “well done” (PT, 21023-22023-1) and “well organized and well laid-out” (CG, 31053-32053-1). A few participants referred to TEMPO as “holistic” (PT 51002-52002, CG 51002-52002-1), as it included information on a range of topics (in one place) and focused on both patients and caregivers. From HCPs’ perspectives, TEMPO was “novel” (HCP-3303) and “an exceptional tool” (HCP-53005). However, there was consensus among HCPs that dyads needed to “invest the time” and “do the work” (HCP-83003). HCPs highlighted that those who “connect” (HCP-53002) with TEMPO and remained “committed” (HCP-53005) would most likely implement the skills learned. All HCPs would recommend TEMPO and use it in routine care.

#### 3.3.1. Online, Self-Directed Format

The self-directed format was overall endorsed. A few participants who had experienced challenges with group or gym-based exercise routines expressed enthusiasm about exercising at their own pace and in the comfort of their own homes. However, a couple of dyads suggested that reminders and progress indicators would support the self-directed format. Although the online delivery of the intervention was acceptable to all dyads, a third of them wanted more options to print the materials. HCPs thought TEMPO was an efficient way to offer services to a larger number of people, and to people who might not have access to psychosocial services. However, they felt that TEMPO would be most appropriate following an in-person needs assessment, or as an additional resource to gain further information on topics discussed during medical consultations.

#### 3.3.2. Dyadic Use

Most of the dyads allocated specific times to work on modules together and went through them sitting in front of the computer (at the same time). These dyads either had both partners entering information, or one participant responding to questions and discussing the choices with the other person sitting nearby. Only one caregiver said that she did not read any of the modules.

The dyadic nature of TEMPO also seemed to help patients and caregivers in coaching each other to achieve their goal. One patient explained: “So let’s say, for example, I have a down day […] she encouraged me to […] just go walk the block or […] do something in the backyard […] just to get some exercise” (PT, 81019-82019-3).

The focus on the dyad was endorsed by HCPs, particularly the focus on addressing sensitive topics (e.g., psychological and sexual health), as dyads may be less likely to introduce these during medical consultations. One HCP (53005) said:

I especially like that it included information not only for the patients but the caregivers and the opportunity for them to participate […] A couple sitting down to complete [the program] and perhaps the question about libido or sexual function would prompt further discussion amongst themselves.

#### 3.3.3. Modules

The dyads felt that the order of the modules was logical and that their length and the information included were appropriate: “[E]ach module […], linked itself to the one before it or linked itself to the two before it, so everything ran pretty evenly from start to finish” (PT, 31054-32054-H). As time progressed, several dyads re-visited modules to refresh their memory, to ensure they were completing tasks properly, and/or to learn about alternative methods to accomplish goals. Half of the dyads said that the modules should be released all at once or that the delay between modules needed to be shorter. One dyad that completed several modules in one sitting explained: “Because I was busy sometimes, we were doing two modules in a row. So, we were sitting down, [partner’s name] and I, on a Saturday or a Sunday at our place up north and we were doing this, right” (PT, 21023-22023-3).

One HCP also emphasized the logical sequence of modules: “I like how it’s broken down into the various modules” (53005). This approach was praised for providing dyads with opportunities to discuss cancer-related issues, and apply the skills taught. In addition, improving the “fluidity between the modules” (HCP-53005) by not forcing the dyads to complete a module before the next one is unlocked was discussed by HCPs.

#### 3.3.4. Online Worksheets

The worksheets most consistently filled out were the needs assessment and the setting of priority needs in Module 1. Most dyads agreed that, despite being long, the needs assessment was relevant and covered their main challenges. Almost all dyads stated that completion of the needs assessment would either be preceded or followed by a conversation on the areas of concern, to share feelings or to discuss what they learned:

We would sit at the computer together and […] sometimes, you know, he’d put down something […] and I’d say to him “Really, is that all you ...” you know or he’d say to me “I didn’t know that was happening” or—so we shared it. We shared our responses and what we were going to say (CG, 31054-32054-1).

Only a couple of dyads had disagreements when completing the needs assessment, and in all instances the final answer prioritized the patients’ wishes. Although many dyads completed the SMARTT goal worksheet for the first goal, it was not consistently completed for the second goal. Despite this, goal setting was identified as one of the most useful aspect of TEMPO:

The SMARTT goals were really good. That was a good thing to work through, and understanding what SMARTT meant, and going through it step by step […]. We did print the action plan and filled it out, and thus far we seem to be on track (CG, 31053-32053-2).

One HCP, however, noted that completing the SMARTT goal worksheet “almost felt like homework. I think the motivated type A personality will do it. […] But then if you’re trying to get the non-motivated person then they’re not going to be motivated” (HCP-53001).

A little more than half of the dyads used the coping plan in Module 3 to overcome setbacks in meeting goals. A similar proportion of dyads completed worksheets in the subsequent modules. Of note, it is not because the dyads did not complete the worksheets online that these were not used. Several dyads preferred to take notes and to print certain pieces of information, whereas a few others chose to complete the worksheets orally (not writing anything down). The majority of dyads used the mood-monitoring tool included in Modules 2–5.

#### 3.3.5. Time Required

The length of time needed to go through modules varied but regardless of the actual time, was deemed appropriate by most. The dyads took anywhere between 10 min to several evenings to complete Module 1; 15 min to several days to complete Module 2; 15 min to two evenings to complete Module 3; 15 min to an hour and a half to complete Module 4; and 15 min to an hour to go through Module 5.

#### 3.3.6. Health Library

Along with the goal setting in Module 2, the health library was one of the most appreciated aspects of TEMPO. All dyads used the health library, to various extents. The dyads were generally satisfied with the clarity, relevance, and comprehensiveness of the information included:

I think being able to access the library is great […] Because that brings all the resources […] to me that’s brilliant, and it should be something that should be pushed out there to the people […] Because it hits a lot of the important emotional points and different stages of learning about cancer, going through deciding what to do, how to deal with it, getting resources, etc. (PT, 11019-12019-2).

Most dyads reported navigating back and forth between the modules and the health library and focused only on the information that was relevant to them. Three sections of the health library were particularly appreciated: communicating with your health care team, supporting one another, and wanting to feel more fit and healthy. However, several dyads revealed that some of the information was not new, albeit it was a good “refresher” (PT, 81019-82019-3). The contents of TEMPO were deemed to be complementary to the information received from HCPs: “Many groups are teaching about exercise, nutrition etc., but I think it being repeated over and over and over again is very good because there’s a little bit of learning at each time” (CG, 31051-32051-2).

The usefulness of information contained in TEMPO was also underscored by HCPs: “[O]verall I think it would be helpful simply because it is a fairly vulnerable population that really doesn’t get enough perhaps attention” (HCP-53002). HCPs spoke of the impressive “breadth” (HCP-53005) of information, and highlighted the value of the information on navigating the health care system and connecting/communicating with HCPs. However, there was some concern that the amount of material could be overwhelming, especially for people with low literacy and lower English proficiency (HCP-83003, HCP-53001). Conversely, there was recognition that TEMPO is an informational resource that could be used in a targeted way: “You can […] pay attention to the things that impact you and ignore the things that don’t” (HCP-53005).

#### 3.3.7. Tailoring

The majority of the dyads felt that TEMPO was generic, and not tailored to their specific needs, but acknowledged that personalizing it would be challenging due to variability in cancer trajectories, age, fitness levels, and personal circumstances. Several dyads felt that TEMPO was more geared towards patients as opposed to caregivers. A few participants observed that TEMPO was not personalized based on treatment trajectory and treatment outcome, and that it was cancer-centric and did not address co-morbidities such as diabetes. Similarly, a few HCPs called for more tailoring of TEMPO.

#### 3.3.8. Pedometers and Resistance Bands

Almost three quarters of dyads referred to using the pedometers and/or resistance bands supplied, at times in combination with other exercises that they had been accustomed to such as weight training. The majority of the dyads tracking their step counts with the pedometers did so to compare their step count to build a healthy competition or to make sure they were meeting their goals. One dyad explained:

When we got the pedometers, we realized that well, a half an hour walk isn’t getting us close to our 10 000 steps. And so, […] we changed our goal really from a half an hour walk to walking to get our 10 000 steps (PT, 31053-32053-3).

#### 3.3.9. Sailing Metaphor

About half of the dyads appreciated the sailing metaphor and argued that it facilitated learning: “I thought the analogy of sailing was really good, because it is like that. It’s kind of, like sometimes you have a smooth ride, sometimes you have a rough ride, sometimes it’s up and down” (CG, 11019-12019-1). Some dyads felt it diminished the seriousness of the illness: “Come on, we got cancer here. We’re dealing with something serious. Let’s not try to make it a vacation” (PT, 11023-12023-1). Others felt that they could not relate to the notion of sailing and would have preferred a camping metaphor. Similarly, one HCP (53005) stated that despite the fact that it made sense to include an analogy, the notion of sailing might be perceived as minimizing what the dyad is going through.

#### 3.3.10. Accessibility of TEMPO

All HCPs recognized the limitations of TEMPO in reaching particular sub-groups, namely, those with low health literacy or who speak a language other than English; those of older age; those with limited computer literacy (*n* = 3); and those patients who are sicker or homeless. None of the participating dyads experienced major challenges in accessing TEMPO, but also acknowledged that some segments of the population might not be able to access TEMPO.

### 3.4. Reported Benefits and Usefulness

The perceived benefits reported by dyads included: increasing PA, enhancing communication, working more together as a dyad, supporting goal fulfillment, managing emotions, increasing knowledge and repertoire of self-management skills, and/or seeking additional support. The majority of the dyads mentioned that they derived at least one of these benefits.

#### 3.4.1. Increasing PA

Most dyads reported increased PA levels: “TEMPO really pushed [us] to consider exercise. Like we always kind of knew, but it kind of focused us on doing it, you know” (CG, 21037-22037-2). Most dyads exercised together:

But I think the good thing, which we’ve enjoyed with TEMPO, is that […] we were always doing things independently, and for the first time in 45 years of marriage, we’re actually doing things together (CG, 21037-22037-2).

Walking was typically the initial exercise, but as time progressed, participants described a wider range of exercises (e.g., strength training, jogging, biking). Almost two thirds of the dyads described challenges to continued PA, including cancer-related obstacles (e.g., needing surgery), lack of motivation or inability to maintain discipline, physical limitations unrelated to cancer, managing conflicting demands of day-to-day life, weather, low energy/too tired, and lack of time. In response to these setbacks, the dyads described active perseverance (e.g., “Substitute activities to something suitable and fits in to weather and time” 31003-32003), sticking with goals, idleness (e.g., taking “time to recover from recent surgery” 11003-12003), getting motivation from their partner, planning to engage in dyadic exercise to maintain accountability, and/or being satisfied with progress despite setbacks. One caregiver explained:

I go out when I feel good enough to go out and I ride my bike when I don’t feel good enough to go out. And I stay home and look after myself otherwise. And I don’t feel badly about that (CG, 31051-32051-3).

Dyads who described high levels of PA prior to TEMPO recognized its benefits in keeping them “disciplined” (21023-22023-3), allowing them to “focus on goals” (31053-32053-3), and “working together a[t] physical activity” (21037-22037-2). Both patients and caregivers further described concrete health benefits as a consequence of their increased PA levels, including improvements in cancer “brain fog” (81019), fewer episodes of incontinence, increased energy level, reduced pain, improved strength, and improved sleep.

#### 3.4.2. Enhancing Communication

Although most dyads did not set out to work on their communication at the beginning of TEMPO, this was a benefit the majority of dyads did not expect. Completing TEMPO helped dyad “focus discussions” (PT, 11019-12019-1), tackle “conversations that were […] a bit uncomfortable” (CG, 21023-22023-1), and gain “insight into each other” (CG, 21037-22037-1). The needs assessment in Module 1 was identified as particularly beneficial in laying the foundation for further communication. Engaging in PA together also seemed to have encouraged communication. As described by one patient: “I would say it’s probably doubled or tripled […] both our exercise and our communication” (PT, 31003-32003-2). The caregivers often recognized this benefit before acknowledging the benefits of PA itself:

We talk more to each when we’re out having a walk than we do if we’re sitting across from each other having supper for example. So for us, walking is probably the best stress reliever, communication means, simply because it seems [we’re] both more at ease when we’re walking (CG, 31054-32054-1).

#### 3.4.3. Working More Together As a Dyad

Aligned with the communication benefit, most dyads emphasized working more together, a benefit that was noted with continued use of TEMPO. For some participants, working together was “the best part of the program” (CG, 11023-12023-2). One dyad explained:

Patient: Because it was important for us to be working together, but —

Caregiver: I think there’s a benefit of a collective […] workload.

Patient: Yeah. If we did it individually, one of us could easily drop off. You know, it’s like an accountability thing (21037-22037-2).

#### 3.4.4. Supporting Goal Fulfillment

Several participants described a process in which TEMPO helped the dyad “focus” or “narrow in” (81020-82020-1) on which aspects of the cancer were most important to address. As described by one patient, “the program’s set up like this, you need to establish a baseline, you need to establish something to focus in on because you can’t focus on everything” (PT, 81019-82019-1). Most dyads spoke of achieving their goals or “moving in the right direction” (PT, 31003-32003-2). One caregiver said: “I think the fact that we had goals to make and that we actually achieved them is a testament to the program” (CG, 11007-12007-3).

#### 3.4.5. Managing Emotions

Several dyads described emotional benefits of participating in TEMPO, including reduced anxiety/depression, improved stress/anger management, and addressing fears surrounding recurrence. One patient explained:

I mean anxiety is awful, it was a new thing for me and there was so much anxiety at the beginning of the study, and it’s not completely gone, but I would say it’s 95% or 90% gone […] this study helped us out and they were putting the emphasis on physical activity to reduce the anxiety and it’s so true (PT, 21023-22023-3).

#### 3.4.6. Increasing Knowledge and Repertoire of Self-Management Skills

TEMPO provided the participants with a centralized repertoire of information that they could consult as needed and apply in a meaningful way to their situation. Slightly more than half of the dyads said they benefited from new or useful information provided by TEMPO. Those participants who had been living with cancer longer, described that the information provided in TEMPO “was review as opposed to new” (PT, 11023-12023-3), and thereby acted as a reminder or a recap (*n* = 11) of information previously received at earlier stages of the cancer trajectory. One patient described that this information equipped him with “some ammunition” to tackle cancer related issues (PT, 81018-82018-3). Learned skills were engaging in PA, coping (e.g., how to overcome challenges and manage stress), SMARTT goal setting, and developing a question prompt list for the oncologist.

#### 3.4.7. Seeking Additional Support

A few dyads described how TEMPO enabled them to acknowledge the need to seek additional support and offered advice on how to do so. One dyad explained:

Caregiver: One thing individually that TEMPO did help me with, [is] it did kind of push me to go the doctor. […] She put me on some antidepressants, a low dose. [...] When I looked at that […] graph, the one where it tells your moods and everything—[…] [I was] feeling overwhelmed, and I went and discussed it with her and they have been almost a life changer. It’s really made a big change in my life […].

Patient: Which has changed my life too (21037-220370-2).

Although most dyads expressed having the necessary support, they appreciated that TEMPO encouraged them to “take stock” (PT, 11007-12007-3) of these resources (particularly in Module 4) and acknowledged their potential usefulness to others:

### 3.5. Improvement Suggestions

The participating dyads and HCPs identified concrete improvements to TEMPO, which are detailed in Table 3. The most common improvements cited included: reduce repetition and condense information in modules, tailor content to patient/caregiver or dyad, more options for exercises or a way to personalize exercise, and more printable/paper-based options for modules.

## 4. Discussion

The results of this qualitative study provide support for the acceptability and satisfaction of TEMPO among prostate cancer dyads. An active engagement with TEMPO provided dyads with the necessary information that enabled them to identify and reflect on their needs and supported dyads as they learned and incorporated new self-management strategies (particularly PA) into their daily routines. Furthermore, the study findings offer preliminary insight into the perceived usefulness of TEMPO. The key findings that will be discussed are: (a) PA is an acceptable self-management strategy for several physical and emotional concerns for both patients and caregivers, (b) most dyads used TEMPO together, (c) the dyads coached each other in achieving their goals, and (d) the dyads perceived TEMPO as useful.

PA was by far the dyads’ preferred self-management strategy, not only for its physical benefits, but also to help in the management of other challenges. Several studies document PA as acceptable to men with prostate cancer [49,50,51]. However, few [52,53,54] have examined its acceptability among caregivers, and the present study contributes to this emerging evidence-base. For instance, one qualitative study on the experience of cancer family caregivers participating in a 6-month PA program described an “upward spiral,” in which caregivers noted “positive ripple effects” from continuous participation in PA (namely allowing them to focus on themselves, increasing energy levels, and improved self-efficacy) [54]. The present study further expands the notion of this “upward spiral” to include an interdependent perspective and highlights the joint benefits for the dyad as a whole.

Traditional models of stress and coping [32] typically focus almost entirely on individual coping strategies. Nonetheless, cancer has been described as a “we-disease” affecting both patients and their families [55]. Research has further documented bidirectional effects of stress and coping within dyads [12,34,56]. As such, interventions must integrate a relational perspective to ensure that dyads address their respective stressors and coordinate their coping efforts [55]. In line with this, TEMPO was designed as a dyadic intervention. Ample research supports dyadic coping interventions that focus on teaching strategies such as maintaining normalcy, cognitive coping strategies, religious/spirituality coping, open communication, joint problem solving, and social support [56,57]. However, the present study adds that PA might be an effective strategy (to date under-studied and under-utilized) to support dyadic coping.

The dyads were not instructed how to use TEMPO, and most decided to sit in front of the computer together. In comparison, a dyadic web-based intervention (CARES) for oral cancer survivors and their caregivers offered an alternative approach, in which separate log-ins and role-tailored content were offered [58]. Nonetheless, both TEMPO and CARES utilized a collaborative approach, and focused on the joint building of self-management skills, encouraging communication within the dyad [58]. This suggests that website features that foster patient–caregiver sharing, offer opportunities to work jointly on assignments, and allow dyads to provide feedback to one another are foundational to enhancing dyadic coping skills. A 2020 literature review of dyadic web-based interventions for cancer patients and their caregivers supports the use of these approaches in achieving small to large positive impacts across physical, emotional, and relational health [59].

Although increased social support helps individuals to make positive behaviour changes [60], most studies on caregiver PA programs target the individual caregiver. This is despite reviews of psychosocial interventions emphasizing that when patients and caregivers (as a dyad) engage in a program, important synergies are achieved that can contribute significantly to each person’s well-being outcomes [17,18]. TEMPO dyads seemed to coach one another as they worked towards attaining their PA objectives. This is in line with previous studies finding that health coaching can enhance the effects of self-directed intervention [61,62]. As part of a 12-week PA intervention for breast and colorectal cancer survivors, Cadmus-Bertram et al. [63] encouraged survivors and their self-selected support partners to meet their PA goals, and highlighted the strategies most often used to accomplish this, including exercising together and phone communication. The results of the present study offer new insight into how dyads may engage in self-coaching and motivational strategies to support one another in meeting PA goals.

According to the Technology Acceptance Model (TAM) [64], the perceived usefulness of an intervention like TEMPO is a key predictor of use. TEMPO dyads described numerous joint benefits, most notably increasing PA and enhancing communication. Although these benefits will be further explored in a subsequent trial, many of the benefits identified by dyads are in line with previous studies [25,26,27]. A recent meta-analysis of e-health interventions for cancer dyads [65] gives further evidence that telehealth interventions have a small (but significant) positive impact on caregivers. However, over 80% of the studies included in this meta-analysis reported on interventions that required practitioner engagement and/or guidance. TEMPO addresses this gap and offers a potential avenue for achieving the desired benefits, while employing a sustainable and economical approach to improving dyadic coping in response to a prostate cancer diagnosis.

Of note, after completing TEMPO, caregivers particularly seemed to value the focus on enhancing positive dyadic coping skills. Caregivers often assessed improvements in PA as a secondary (but complementary) benefit to the communication benefit. This is in line with Fife and colleagues [66] who found that caregiver-related coping was most strongly associated with positive dyadic adjustment to a cancer diagnosis (for both the patient and the caregiver). Studies support that poor dyadic coping has a greater impact on caregivers compared to patients, with more caregivers identifying cancer diagnosis as being highly intrusive, and experiencing significant cancer-related distress or burden [67,68].

Finally, the dyads emphasized the usability of the TEMPO platform. Most participants felt that the platform was user-friendly and well organized, despite describing normal setbacks typically associated with learning to navigate a new interface. Nonetheless, dyads and HCPs alike generated a comprehensive list of suggested improvements, such as improving site navigation, improving the goal setting functions, and modifications to the lock-unlock feature of modules. Phase II of TEMPO consists of a multicentre, stratified, parallel, two-group pilot randomized control trial. Recruitment is ongoing, and concrete improvements to the TEMPO interface have been made based on these suggestions. Notably a “print feature” has been added, and modules become immediately available upon completion of the preceding module to further increase usability.

### Study Limitations

We used rigorous data collection and analyses approaches. The majority of patients and caregivers in our sample were over the age of 60, retired, capable of engaging in medium to high intensity physical activity, and reporting supportive dyadic relationships. Therefore, more studies with younger, employed, less fit sub-groups, and those dyads who experience less positive dyadic coping are needed. Also, we did not measure digital literacy and it is not known the extent to which the sample was comfortable using a computer. In addition, in this first, acceptability phase, TEMPO was only available in English. Despite the fact that our sample included bilingual Francophones and other linguistically diverse participants, more data are required to assess the acceptability of TEMPO in patients and caregivers who do not understand English.

## 5. Conclusions

Given the critical role that caregivers play in maintaining patients’ health and wellbeing, it is imperative that more studies test effective and sustainable methods to preserve or improve their QOL. Although the number of web-based, self-management interventions is growing, TEMPO is the first one specifically designed to address the needs of both patients and their caregivers; and thus fills a niche for caregivers who find it difficult to participate in programs with more structured requirements. TEMPO targets multiple risk factors to improve dyadic coping and combines the best evidence in terms of PA training and self-management. The participating prostate cancer patients, their family caregivers, and members of the health care community were in agreement that TEMPO has great potential in addressing the physical, emotional, and psychosocial challenges that accompany a cancer diagnosis; and that it achieves this in a way that facilitates translation into practice. This study has contributed to developing the knowledge base on TEMPO’s acceptability and usefulness that will directly contribute to the design of a larger trial.

## Figures and Tables

**Table 1:** Socio-demographic data for participant dyads (*n* = 19).

Socio-Demographic Characteristic	Caregivers (*n* = 17) * N (%)	Patients (*n* = 19) N (%)
Age (Years)		
40–49	1 (5.9%)	1 (5.3%)
50–59	4 (23.5%)	2 (10.5%)
60–69	9 (52.9%)	11 (57.9%)
≥70	3 (17.7%)	5 (26.3%)
Mean	62.3	64.7
SD **	6.62	7.22
Median	63	66
Language		
English	15 (88.2%)	16 (18.2%)
French	1 (5.9%)	1 (5.3%)
Other	1 (5.9%)	2 (10.5%)
Education		
High School	2 (11.8%)	1 (5.3%)
Post Secondary diploma	4 (23.5%)	7 (36.8%)
Undergraduate degree	11 (64.7%)	6 (31.6%)
Masters Degree	0 (0%)	3 (15.8%)
Doctorate Degree	0 (0%)	2 (10.5%)
Health Conditions		
Arthritis	6 (35.3%)	6 (31.6%)
Diabetes	2 (11.8%)	7 (36.8%)
Emotional problems	5 (29.4%)	5 (26.4%)
Hypertension	4 (23.5%)	9 (47.4%)
Heart Problems	1 (5.9%)	5 (26.4%)
Intestinal polyps	1 (5.9%)	3 (15.8%)
Liver disease	1 (5.9%)	2 (10.5%)
Sleep Apnea	1 (5.9%)	5 (26.4%)
Stomach problems	2 (11.8%)	3 (15.8%)
Thyroid	2 (11.8%)	3 (15.8%)
Household Income (*n* = 19 Dyads)		
40,000$ to 59,999$	3 (15.8%)	
60,000$ to 79,999$	1 (5.3%)	
80,000$ to 99,999$	4 (21.0%)	
≥100,000$	7 (36.8%)	
Prefer not to answer	4 (21.0%)	
Marital Status (*n* = 19 Dyads)		
Single	1 (5.3%)	
Married	17 (89.4%)	
Common Law	1 (5.3%)	
**Patient Cancer Characteristics**		
Years since Prostate Cancer Diagnosis		
<1 year		4 (21.0%)
1–3 years		10 (52.6%)
3–5 years		5 (26.4%)
Stage of Cancer at Diagnosis		
Early Stage		11 (57.9%)
Advanced Stage		6 (31.6%)
Unknown		2 (10.5%)
**Cancer treatments receiving, received, or plan to receive**		
Surgery		12 (63.2%)
Chemotherapy		4 (21.0%)
Radiotherapy		10 (52.6%)
Hormone Treatment		7 (36.8%)
Brachytherapy		2 (10.5%)
Watchful waiting		2 (10.5%)
Other		1 (5.3%)

* Caregiver data only available for 17/19 dyads. One patient was interviewed alone (his caregiver participated in TEMPO but not in the interviews), while the remaining caregiver data are missing. ** Standard deviation.

**Table 2:** Physical activity (PA) undertaken by dyads, patients, and caregivers during TEMPO.

Patient and Caregiver Together (14/19 (74%) Dyads Reported Engaging in PA Jointly, with Several Dyads Engaging in More than One Activity)	Patient Only (13/19 (68%) Patients Reported Engaging in PA Independently, with Several Patients Engaging in More than One Activity)	Caregiver Only (5/18 (28%) Caregivers Reported Engaging in PA Independently, with Several Caregivers Reporting Engaging in More than One Activity)
Walking, including dog walking (*n* = 12)	Walking (*n* = 3)	Group fitness classes (spinning and weight training) (*n* = 2)
Resistance training (*n* = 3)	Resistance training (*n* = 3)	Gym training (*n* = 1)
Weight and strength training (*n* = 2)	Home exercises, including TRX * (*n* = 3)	Squash (*n* = 1)
Working on property, gardening (*n* = 2)	Skiing (*n* = 2)	Yoga (*n* = 1)
Home gym training, including elliptical machine (*n* = 1)	Group fitness classes (*n* = 2)	Swimming (*n* = 1)
Hiking (*n* = 1)	Weight training (*n* = 1)	Hiking (*n* = 1)
Horseback riding (*n* = 1)	Cycling (*n* = 1)	Walking (*n* = 1)
Cycling (*n* = 1)	Swimming (*n* = 1)	Golf (*n* = 1)
Swimming (*n* = 1)	Pilates (*n* = 1)	Home gym training (*n* = 1)
Golf (*n* = 1)	Gym training (*n* = 1)	
Relaxation exercises (*n* = 1)	Stretching (*n* = 1)	
**Totals:**	**Totals:**	**Totals:**
1 activity—7 dyads 2 activities—3 dyads 3 activities—3 dyads 4 activities—1 dyad	1 activity—9 patients 2 activities—3 patients 4 activities—1 patient	1 activity—2 caregivers 2 activities—2 caregivers 4 activities—1 caregiver

* The TRX System, also known as Total Resistance exercises, refers to a specialized form of suspension training.

**Table 3:** Suggested Improvements to TEMPO.

Categories of Proposed Changes	Patients/Caregivers	HCPs *
**Navigation**	Improve site navigation (*n* = 4) Improve navigation in the library/finding content (*n* = 2)	
**Content**	Reduce repetition/condense information (*n* = 10) Tailor content (*n* = 10) More detail/depth in modules (*n* = 2) Precise questions in needs assessment (*n* = 2) Tone of TEMPO is that of a study (*n* = 2), make it more interactive (*n* = 1) Reduce the number of worksheets (*n* = 1) TEMPO available in other languages (*n* = 1) Emphasize what could be done after TEMPO (*n* = 1)	Reduce repetition/condense information (*n* = 2) Tailor content (*n* = 1) SMART goals too onerous (*n* = 1) Information must be consistent with practices (*n* = 1)
**Additional sections**	Overview of modules and an index page (*n* = 3) Introductory section outlining goals and benefits of TEMPO (*n* = 2) TEMPO FAQ ** section (*n* = 1) Better instructions on how to set TEMPO on computer (*n* = 1) Clearly state purpose of worksheets (*n* = 1)	Explain the cancer journey and how TEMPO fits into it (*n* = 1) Include a glossary of terms used in each module (*n* = 1)
**Additional information**	More options of exercises or a way to personalize exercise, based on physical abilities (*n* = 7) Nutrition information (*n* = 3) Managing emotions (*n* = 2) Epidemiological data on prostate cancer (*n* = 2) Testimonials and tips from people who have had success achieving goals (*n* = 2) Add more visual information (*n* = 2) Acknowledge co-morbidities (*n* = 1) Strategies to deal with brain fog (*n* = 1) Dyadic communication tips (*n* = 1) How to deal with erectile dysfunction (*n* = 1) Weight loss guidance (*n* = 1) Information on undesired weight loss (*n* = 1) Physical balance information (*n* = 1) Communicating with family and friends (*n* = 1) Tips on increasing social circle, not necessarily with other people living with cancer (*n* = 1) Reflect age diversity in videos (*n* = 1) Public figures survivor testimonials (*n* = 1) More detail on how to perform certain exercises (*n* = 1) State the purpose of proposed activity, e.g., “to help with abdominal strength” (*n* = 1) Allow to register information on other physical activities (*n* = 1)	Disease, symptom management, treatment, palliative care and advance care planning, applicable to all stages (post-treatment, metastatic, end of life) (*n* = 3) Sexual health (*n* = 2) Navigate the health care system (*n* = 2) Available trials (*n* = 1) Pictures, graphs and other visual elements (*n* = 1) Ensure diversity in photos (*n* = 1)
**Technical features**	Improve goal setting functions (*n* = 21) More printable/paper-based options for modules (*n* = 6) Tools to track progress (*n* = 4) Incorporate partner’s needs more explicitly, e.g., by providing more options for goal-setting (*n* = 3) Encourage to persevere with goals (*n* = 2) Allow to work on other goals, if initial ones achieved (*n* = 1) Push only relevant library content (*n* = 1) Allow for online completion of forms (*n* = 1) Provide more space to enter information about barriers (*n* = 1) Incorporate a quiz at the end of modules (*n* = 1) Make TEMPO easy to find on the web (*n* = 1)	Incorporate a way to encourage, coach and touch base (*n* = 2) Develop a mobile TEMPO app (*n* = 1) Offer an “express” version of TEMPO (*n* = 1) Add a search function for health library (*n* = 1) Allow for online completion of forms (*n* = 1) Implement a way to track progress electronically or on paper (*n* = 1) Fix broken links and text that is cut in some handouts (*n* = 1)
**Accessories**	Send accessories at the start of the program, otherwise difficult to incorporate them into goal setting (*n* = 1) Send two sets of elastic bands (*n* = 1) Display on pedometers very small (*n* = 1) Pedometers only keep a day’s worth of information (*n* = 1) Include information on how to start using them (*n* = 1)	
**Lock-unlock modules**	Release all modules at once (*n* = 4) or shorten gap between modules (*n* = 4) Make explicit the delay between modules (*n* = 2) Include reminders (*n* = 2)	
**Introduction and endorsement**	TEMPO needs to be promoted/introduced by HCPs (*n* = 1)	TEMPO needs to be introduced and explained by HCPS (*n* = 2) Introduce TEMPO *via* patient and family education programs (*n* = 2) Endorsements from physicians (*n* = 1) Make it explicit that it is a reputable site used across Canada (*n* = 1) Assess the participants’ knowledge and if able to utilize it (*n* = 1) Program support person should be available to participants as they go through it (*n* = 1)
**Peer support**	Opportunity to obtain feedback, ask a question or engage in group discussion (*n* = 2) Pair struggling participants with those who have succeeded (*n* = 1) Parts offered through group gym activities (*n* = 1) Trained volunteers available to talk if a participant has a question (*n* = 1)	Incorporate a way to connect with other patients and their families (*n* = 1)
**Additional resources**	Referrals to relevant and functioning social support resources, separate support resources for caregivers (*n* = 2) Referrals to other relevant websites (*n* = 2)	Referrals to support services (*n* = 1) Encourage participants to contact the services offered in their cancer centers (*n* = 1)

Note: * Health Care Professionals (HCPs); ** Frequently Asked Questions (FAQs).

## Supplementary Materials

The following are available online at https://www.mdpi.com/2077-0383/9/10/3284/s1. Supplementary data 1: Figure S1. TEMPO Health Library landing page. This illustrates the different topics and organization of information contained in the health library; Table S1. TEMPO Health Library Topics of the 45 Factsheets; Supplementary data 2: Interview Guides used in the Qualitative Evaluation of TEMPO.
